# An Implementation of the Fundamental Parameters Approach for Analysis of X-ray Powder Diffraction Line Profiles

**DOI:** 10.6028/jres.120.014

**Published:** 2015-10-21

**Authors:** Marcus H. Mendenhall, Katharine Mullen, James P. Cline

**Affiliations:** 1 National Institute of Standards and Technology, Gaithersburg, MD 20899 USA; 2 University of California Los Angeles, Department of Statistics, 8125 Math Sciences Bldg., Los Angeles, CA 90095-1554 USA

**Keywords:** Fundamental Parameters Approach, powder diffraction, Python, Standard Reference Material, X-ray line profile

## Abstract

This work presents an open implementation of the Fundamental Parameters Approach (FPA) models for analysis of X-ray powder diffraction line profiles. The original literature describing these models was examined and code was developed to allow for their use within a Python based least squares refinement algorithm. The NIST interest in the FPA method is specific to its ability to account for the optical aberrations of the powder diffraction experiment allowing for an accurate assessment of lattice parameter values. Lattice parameters are one of the primary certified measurands of NIST Standard Reference Materials (SRMs) for powder diffraction. Lattice parameter values obtained from analysis of data from SRMs 640e and 660c using both the NIST FPA Python code and the proprietary, commercial code Topas, that constitutes the only other actively supported, complete implementation of FPA models within a least-squares data analysis environment, agreed to within 2 fm. This level of agreement demonstrates that both the NIST code and Topas constitute an accurate implementation of published FPA models.

## 1. Introduction

**Fig. 1:**
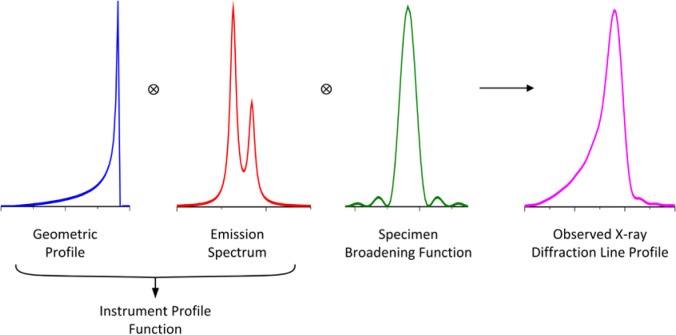
Generation of a line profile via convolutions in the FPA.

Measured X-ray powder diffraction line profiles are affected by the geometry of the diffractometer, the shape of the X-ray emission spectrum and the physical characteristics of the sample as shown diagrammatically in Fig. 1. Certain aberrations embodied in the geometric instrument profile are highly asymmetric and will displace the observed position of profile maxima; therefore, these maxima are not indicative of the true lattice spacing. Furthermore, the level and direction of the displacement can vary dramatically as a function of diffraction angle. High-accuracy determination of the crystallite lattice spacing from measured line profiles requires accounting for such effects in the model for line profile shape. The Fundamental Parameters Approach (FPA) for the description of X-ray line profiles is a convolution-based line profile modeling method that describes the measured line profiles as the convolution of peak profiles representing the emission spectrum with a number of aberration functions, each representing a certain aspect of the instrument configuration or sample microstructure that affects the measured peak position and/or shape. The parameters of FPA models are entirely interpretable in terms of the physical characteristics of the underlying experimental set-up. Non-physical parameters obtained in an FPA analysis can be used as a “feedback loop” in discerning difficulties with regards to the experimental setup [1].

The FPA is the primary means by which X-ray line profiles are analyzed in the development of National Institute of Standards and Technology (NIST) Standard Reference Materials (SRMs) for powder diffraction. The SI traceability of the lattice parameter measurement is established through the characterization of the Cu *K_α_* emission spectrum as provided by Hölzer [2]. The FPA models used for powder diffraction patterns were developed initially by Wilson [3], and in essentially modern form by Cheary and Coelho [4–6]. Later additions included new models and corrections [7, 8]. One of the first publicly available codes to offer the FPA capability was Xfit, followed by Koalariet [9, 10]. Shortly after these two public domain codes ceased to be supported, the commercial product, Bruker Topas [11]^1^, was released, continuing with the same FPA formalism that had been established with the previous codes. With the use of Topas for SRM certification, commencing with SRMs 660a [12] and 640c [13], numerous self-consistency studies were performed that indicated the FPA models within Topas were operating in accordance to expectations [8]. However, Topas is a proprietary code; a quantitative means to verify that its operation was in adherence with published FPA models was the development of an independent code written directly from the examination of said FPA models.

In this work, we present a robust set of numerical methods by which computations required for the FPA can be carried out. An implementation of the algorithms that are described, written in the Python [14] programming language, the NIST Fundamental Parameters Approach Python code (FPAPC), is provided as supplementary material^2^. We make no attempt to repeat any of the theory or background presented in [4, 5, 8]; the focus is on clear and efficient implementation and verification. We introduce one new FPA model, for the defocusing across the face of a silicon-strip position-sensitive detector (Si PSD) in Sec. 2.5.7. All of the convolutions are carried out via multiplication in Fourier space, per the convolution theorem (see Appendix A, Sec. 5). As such, the emission spectrum and all of the aberrations are directly computed in Fourier space. The exceptions are the axial divergence and the flat-specimen models; these are computed in real space and then transformed into Fourier space. However, this approach leads to the periodicity implicit in Fourier methods that distorts the function calculations at the boundaries; we therefore describe in Sec. 2.6 a method to correct said periodicity errors. The organization and combination of parameters in this work, especially with respect to line shape and crystal size, is entirely for computational expediency, and does not reflect any physical relationship between these quantities.

## 2. Components of the Fundamental Parameters Model

### 2.1 Definitions and Notation

*L_s_* the length of the sample in the axial direction (perpendicular to the diffraction plane)
*L_x_* the length of the X-ray source in the axial direction
*L_r_* the length of the receiver slit in the axial direction
*R* the radius of the diffractometer, with the assumption of a symmetrical system
2*θ* the detector angle (twice the diffraction angle)
Ω the specimen angle
*β* the angle of a ray of X-rays off the equatorial plane (in the axial direction)
*W* the full width, in 2*θ* space, of the window over which a peak is being computed
2*θ*_0_ the center of the computation window
*N*_2_*_θ_* the number of bins in the computation window
$\vec{\varepsilon }[i]$ the *i*th element of array $\vec{\varepsilon }$, with $\vec{\varepsilon }[0]$ being the first element
$\vec{\varepsilon }[-i]$ the *i*th element from the end of array $\vec{\varepsilon }$, so $\vec{\varepsilon }[-i]$ is the last element
$\vec{\varepsilon }[i:j]$ the elements of an array $\vec{\varepsilon }$ with indices between *i* and *j*−1 (inclusive), so it has length *j*−*i*
$\vec{\varepsilon }+x$ an operation between an array and a scalar operates element-by-element on the array with the scalar
$\vec{\varepsilon }+\vec{x}$ an operation between two arrays is done element-by-element
$f(\vec{\varepsilon })$ a function applied to an array is an array of the same length with the function applied to each element
**#** in pseudo-code sections, everything after this on a line is a comment
**scaling** we will present all equations below in a manner that is mostly compatible with the usage established by Topas. Lorentzian widths and Gaussian widths are expressed as the full-width at half-maximum (FWHM) of the peak shape. However, all lengths are uniformly scaled; any consistent unit of length can be used, but all lengths must be the same units. The reference code we provide takes all angles in degrees, and converts them internally to radians.

### 2.2 Initialization of Parameters

To start a calculation, we assume that the result will be a peak shape, uniformly gridded in 2*θ* space, centered at 2*θ*_0_, with *N*_2_*_θ_* points (which will typically be even and either a power of two or, for modern Fast Fourier Transforms (FFT) packages, products of powers of 2, 3, 5, and 7, typically), and with full width *W*. To match the standard of typical Fourier transform package implementations, using a common convention for transforms of purely real data sets, we will carry only the coefficients corresponding to non-negative angular frequencies. We will assume the length of the $\vec{\omega }$ array is *N*_2_*_θ_*/2 +1, in which the highest frequency cosine component is put at the end, rather than folded back into the zero-frequency bin as is the result of fuu, complex FFTs. The $\vec{\omega }$ array is initialized, then, to

$$\vec{\omega }[j]=j\frac{2\pi }{W}$$

where *W* is in radians.

### 2.3 Source Spectrum and Crystal Size

We handle the convolutions due to the emission spectrum and due to the crystal size parameters first, and together, because it is numerically efficient to do so. We initialize the Fourier transform buffer $\tilde{F}$ that will be multiplied with all the other convolutions with the sum of the transforms of the emission spectrum broadened by the peak size. These two are kept together because they each have Lorentzian and Gaussian components that are easily combined. It is important to note that these crystallite size contributions have nothing to do with the actual emission spectrum; the grouping is purely historical and convenient.

For an emission spectrum which is a set of *n_λ_* lines, indexed by *k*, each with Lorentzian FWHM *l_k_*, Gaussian FWHM *g_k_*, intensity *I_k_*, and wavelength *λ_k_*, and a Gaussian crystal size component *S_G_* and a Lorentzian crystal size component *S_L_*, and a reference wavelength *λ*_0_ which is typically that of the strongest line in the spectrum, we define:

$$\begin{array}{l} h_{k}=\frac{l_{k}\tan\theta_{0}}{\lambda_{k}}+\frac{\lambda_{k}}{\left(\frac{\pi }{2}S_{L}\right)\cos\theta_{0}}\\ s_{k}^{2}={\left(\frac{2g_{k}\tan\theta_{0}}{\lambda_{k}}\right)}^{2}+{\left(\frac{\lambda_{k}}{S_{G}\cos\theta_{0}}\right)}^{2}\\ {\vec{a}}_{k}=-\vec{\omega }h_{k}\\ \sigma_{k}^{2}=\frac{s_{k}^{2}}{8\log2}\\ {\vec{b}}_{k}=-\frac{{\vec{\omega }}^{2}\sigma_{k}^{2}}{2}\\ {\vec{\delta }}_{k}=\vec{\omega }\left(2\arcsin\frac{\lambda_{k}\sin\theta_{0}}{\lambda_{0}}-2\theta_{0}\right). \end{array}$$ (1)

Then the transform buffer $\tilde{F}$ is initialized via:

$$\tilde{F}=\sum_{k=1}^{n_{\lambda }}I_{k}\exp\left[{\vec{a}}_{k}+{\vec{b}}_{k}-i{\vec{\delta }}_{k}\right].$$ (2)

Care must be exercised with respect to the sign on the complex term in the exponential. Some Fourier transform packages define this differently. It is suggested that if a code is written, the order of the lines in the spectrum be verified. If the spectrum is backwards in the diffraction pattern, this sign should be switched.

### 2.4 Axial Divergence

The axial divergence model of Cheary and Coelho [5] (referred to as the “full” axial divergence model in Topas) is effective for the computation of this complicated function for a wide variety of situations. However, it is very intricate, involving many tests for various boundary conditions, and if implemented without attention to numerical issues can be computationally expensive. We present an implementation that is functionally equivalent, but has many of the boundary conditions clarified, and which is less subject to numerical issues than a literal implementation would be. In particular, we handle the $1/\sqrt{x}$ divergences so that the function can be evaluated more coarsely without loss of smoothness.

For the purposes of a practical implementation of this model, we need to be able to calculate *I*_2_(*β*, *ε*) per Sec. 4.2 of [5] and then, ultimately, the full intensity profile including the effects of Soller slits via integration of their equation 27. The remainder of this section will present this in detail, concentrating on a clean algorithmic implementation, with all theoretical details being referred back to [5, 8].

The first step of the calculation is to set up some parameters that will be needed (with locations in [5] where appropriate):

$$\beta_{1}=\frac{L_{s}-L_{x}}{2R}\;(\text{after eq}.\;15)$$ (3)

$$\beta_{2}=\frac{L_{s}+L_{x}}{2R}\;(\text{after eq}.\;15,\;\text{correcting error in original})$$ (4)

$$\varepsilon_{0}\frac{\beta^{2}}{2}\tan2\theta \;(\text{after eq}.\;26)$$ (5)

$$\varepsilon_{A}=\frac{\cot2\theta }{2R^{2}}\;(\text{a constant we will need}).$$ (6)

Then, following eqs. 15a, b, c, d of [5], we compute the parameters $Z_{0}^{+}$ and $Z_{0}^{-}$ conditionally on *β*:

$$\begin{array}{l} Z_{0}^{+}=\{\begin{array}{cc} \frac{L_{x}}{2}+\beta R(1+\sec2\theta ) & -\beta_{2}<\beta <+\beta_{1}\\ \frac{L_{s}}{2}+\beta R\sec2\theta & +\beta_{1}<\beta <+\beta_{2} \end{array}\\ Z_{0}^{-}=\{\begin{array}{cc} -\frac{L_{s}}{2}+\beta R\sec2\theta & -\beta_{2}<\beta <-\beta_{1}\\ -\frac{L_{x}}{2}+\beta R(1+\sec2\theta ) & -\beta_{1}<\beta <+\beta_{2} \end{array}. \end{array}$$ (7)

Then, from eqs. 18a, b of [5] (with corrections to 18b),

$$\begin{array}{l} \varepsilon_{1}^{+}=\varepsilon_{0}-\varepsilon_{A}{\left(\frac{L_{r}}{2}-Z_{0}^{+}\right)}^{2}\\ \varepsilon_{1}^{-}=\varepsilon_{0}-\varepsilon_{A}{\left(\frac{L_{r}}{2}-Z_{0}^{-}\right)}^{2}(\text{note correction from original})\\ \varepsilon_{2}^{+}=\varepsilon_{0}-\varepsilon_{A}{\left(\frac{L_{r}}{2}-Z_{0}^{-}\right)}^{2}\\ \varepsilon_{2}^{-}=\varepsilon_{0}-\varepsilon_{A}{\left(\frac{L_{r}}{2}-Z_{0}^{+}\right)}^{2}(\text{note correction from original}) \end{array}$$ (8)

but noting the following reordering if 2*θ* > 90°: the *ε*^+^ values get swapped with the corresponding *ε*^−^ values, i.e. $\varepsilon_{1}^{+}⇌\varepsilon_{1}^{-}$ and $\varepsilon_{2}^{+}⇌\varepsilon_{2}^{-}$.

The problem is then divided into two major domains, each with three minor ones. This is most easily done following Table 1 of [5]. This has been amended to include omitted reflections for formulas when 2*θ* > 90°. We present this in two formats: a pseudocode block in algorithm 1, and Table 1.

**Algorithm 1:** Selection of computation boundaries and *β* ranges

if $L_{r}>Z_{0}^{+}-Z_{0}^{-}$: # wide receiver slit
if $Z_{0}^{+}\leq \frac{L_{r}}{2}$ and $Z_{0}^{-}\geq \frac{L_{r}}{2}$:
# parabola apexes entirely within slit
*rr* = 1; $\varepsilon_{a}=\varepsilon_{1}^{+}$; $\varepsilon_{b}=\varepsilon_{2}^{+}$; $\varepsilon_{c}=\varepsilon_{1}^{-}$; $\varepsilon_{d}=\varepsilon_{2}^{-}$
seel if $\left(Z_{0}^{+}>\frac{L_{r}}{2}\;\text{and}\;Z_{0}^{-}<\frac{L_{r}}{2}\right)$or $\left(Z_{0}^{+}>-\frac{L_{r}}{2}\;\text{and}\;Z_{0}^{-}<-\frac{L_{r}}{2}\right)$:
# one apex outside of slit
*rr* = 2; $\varepsilon_{a}=\varepsilon_{2}^{+}$; $\varepsilon_{b}=\varepsilon_{1}^{+}$; $\varepsilon_{c}=\varepsilon_{1}^{-}$; $\varepsilon_{d}=\varepsilon_{2}^{-}$
else:
# both apexes outside of slit
*rr* = 3; $\varepsilon_{a}=\varepsilon_{2}^{+}$; $\varepsilon_{b}=\varepsilon_{1}^{+}$; $\varepsilon_{c}=\varepsilon_{1}^{-}$; $\varepsilon_{d}=\varepsilon_{2}^{-}$
else: # narrow receiver slit
if $Z_{0}^{+}\geq \frac{L_{r}}{2}\;\text{and}\;Z_{0}^{-}\leq -\frac{L_{r}}{2}$:
# parabola apexes hanging off both ends of slit
*rr* = 1; $\varepsilon_{a}=\varepsilon_{1}^{-}$; $\varepsilon_{b}=\varepsilon_{2}^{+}$; $\varepsilon_{c}=\varepsilon_{1}^{+}$; $\varepsilon_{d}=\varepsilon_{2}^{-}$
else if $\left(Z_{0}^{+}>\frac{L_{r}}{2}\;\text{and}\;-\frac{L_{r}}{2}<Z_{0}^{-}<\frac{L_{r}}{2}\right)$ or $\left(-\frac{L_{r}}{2}<Z_{0}^{+}<\frac{L_{r}}{2}\;\text{and}\;Z_{0}^{-}<-\frac{L_{r}}{2}\right)$ :
# one apex of beam within slit
*rr* = 2; $\varepsilon_{a}=\varepsilon_{2}^{+}$; $\varepsilon_{b}=\varepsilon_{1}^{-}$; $\varepsilon_{c}=\varepsilon_{1}^{+}$; $\varepsilon_{d}=\varepsilon_{2}^{-}$
else:
*rr* = 3; $\varepsilon_{a}=\varepsilon_{2}^{+}$; $\varepsilon_{b}=\varepsilon_{1}^{-}$; $\varepsilon_{c}=\varepsilon_{1}^{+}$; $\varepsilon_{d}=\varepsilon_{2}^{-}$

#### 2.4.1 Computing Components of Axial Divergence Shape for Fixed *β*

Using the parameters from algorithm 1 or Table 1, we need to set up the equations from Table 1 of [5] for *F*_1_, *F*_2_, *F*_3_, and *F*_4_. These will be used exactly as defined in the original work, with *r_β_* defining the *β* range to select the equations.

However, this is the component of the computation where the most critical numerical issues must be addressed. First, we assume that the angle offset *ε* is defined on a uniform grid centered at 0 and running from −*w* to *w* where *w* is the half-width of a window on which the axial divergence function in being computed. It will be treated as an array $\vec{\varepsilon }$ and stored as an array of *n* elements. All of the *F* functions compute pieces of a function $y_{0}+k/\sqrt{\left|\varepsilon -\varepsilon_{0}\right|}$. This computation may well include the endpoint where *ε* = *ε*_0_, where this function diverges. Also, due to the discrete binning of *ε*, it may include a point at which the argument (without the absolute value) is in fact negative, but should be truncated to 0. Further, due to the discrete binning, the sum of all of the sampled values of the function may not add up to the integral of the function over the bounds, resulting in inaccuracies in the total X-ray peak intensity, especially in the case of sampling on a relatively coarse grid for computational speed. Finally, again due to discrete sampling, the first moment (centroid) of the distribution may not be exactly correct. This is particularly critical, since shifts in this result in inaccuracy of peak positions. Such shifts can be reduced by using finer computational grids, but this approach is very inefficient. Therefore, we adjust the result so that, in all cases, it has the exact centroid one would expect in the continuum limit.

All of these issues can be addressed with a single ‘helper’ function *F*_0_ which will be used to construct *F*_1,2,3,4_ in a unified manner. This is presented in the next section.

#### 2.4.2 Helper Function *F*_0_

This function will take as formal arguments

- $\vec{\varepsilon }$, the grid of angle offsets on which the results are computed
- buffer $\vec{accum}$ with the same number of elements as $\vec{\varepsilon }$, and each bin will correspond to the line profile at the corresponding angle offset
- *ε*_0_, the position of the singularity
- *ε*_inner_, the boundary closest to *ε*_0_
- *ε*_outer_, the boundary further from *ε*_0_
- *k*, the scale factor
- *y*_0_, the offset from zero

The helper function will sum values of the $y_{0}+k/\sqrt{\left|\varepsilon -\varepsilon_{0}\right|}$ into $\vec{accum}$ bins corresponding to *ε* between *ε*_inner_ and *ε*_outer_, and assure all the numerical issues are dealt with. It correctly integrates up to the singularity, and also assures that the area and centroid of the returned function are accurate, in spite of the sampling of a continuous function onto a discrete grid. It will return the bin indices corresponding to the lowest bin and (highest bin)+1 it actually modified, so that other parts of the computation can work efficiently only on the non-zero parts of $\vec{accum}$. Because of the intricacy of this function, and the need to describe it algorithmically, the details of it are in the Python implementation of the FPA provided as ancillary materials with this paper. The function ’axial_helper’ in the python code is the implementation of *F*_0_.

#### 2.4.3 Computing Complete Axial Divergence for Fixed *β*

With the assistance of the function in 2.4.2, the bookkeeping in Table 1 of [5] is straightforward to implement. The procedure for doing this is shown as pseudo-code in 2.4.4. When this process is complete, one has in hand the arrays representing the functions $I_{2}^{+}$ and $I_{2}^{-}$ for a given *β* and 2*θ*_0_.

#### 2.4.4 Carrying Out Table 1 Computation for *I*_2_

We define the various functions needed for Table 1 in terms of the helper *F*_0_. Note that *F*_0_ modifies the $\vec{dst}$ argument in place, and returns the lower and upper bounds of the part of the array which is modified.

$$\begin{array}{c} F_{1}(\vec{dst},\varepsilon_{\text{out}},\varepsilon_{\text{in}},\varepsilon_{a},\varepsilon_{b})\equiv F_{0}\left(\vec{2\theta }-2\theta_{0},\vec{dst},\varepsilon_{\text{in}},\varepsilon_{\text{out}},\varepsilon_{0},\sqrt{\left|\varepsilon_{0}-\varepsilon_{b}\right|}-\sqrt{\left|\varepsilon_{0}-\varepsilon_{a}\right|},0\right)\\ F_{2}(\vec{dst},\varepsilon_{\text{out}},\varepsilon_{\text{in}},\varepsilon_{a})\equiv F_{0}\left(\vec{2\theta }-2\theta_{0},\vec{dst},\varepsilon_{\text{in}},\varepsilon_{\text{out}},\varepsilon_{0},+\sqrt{\left|\varepsilon_{0}-\varepsilon_{a}\right|},-1\right)\\ F_{3}(\vec{dst},\varepsilon_{\text{out}},\varepsilon_{\text{in}},\varepsilon_{a})\equiv F_{0}\left(\vec{2\theta }-2\theta_{0},\vec{dst},\varepsilon_{\text{in}},\varepsilon_{\text{out}},\varepsilon_{0},+\sqrt{\left|\varepsilon_{0}-\varepsilon_{a}\right|},+1\right)\\ F_{4}(\vec{dst},\varepsilon_{\text{out}},\varepsilon_{\text{in}},\varepsilon_{a})\equiv F_{0}\left(\vec{2\theta }-2\theta_{0},\vec{dst},\varepsilon_{\text{in}},\varepsilon_{\text{out}},\varepsilon_{0},-\sqrt{\left|\varepsilon_{0}-\varepsilon_{a}\right|},+1\right) \end{array}$$ (9)

or, equivalently:

$$\begin{array}{c} F_{1}(\vec{dst},\varepsilon_{\text{out}},\varepsilon_{\text{in}},\varepsilon_{a},\varepsilon_{b})\equiv F_{0}\left(\vec{2\theta },\vec{dst},\varepsilon_{\text{in}}+2\theta_{0},\varepsilon_{\text{out}}+2\theta_{0},\varepsilon_{0}+2\theta_{0},\sqrt{\left|\varepsilon_{0}-\varepsilon_{b}\right|}-\sqrt{\left|\varepsilon_{0}-\varepsilon_{a}\right|},0\right)\\ F_{2}(\vec{dst},\varepsilon_{\text{out}},\varepsilon_{\text{in}},\varepsilon_{a})\equiv F_{0}\left(\vec{2\theta },\vec{dst},\varepsilon_{\text{in}}+2\theta_{0},\varepsilon_{\text{out}}+2\theta_{0},\varepsilon_{0}+2\theta_{0},+\sqrt{\left|\varepsilon_{0}-\varepsilon_{a}\right|},-1\right)\\ F_{3}(\vec{dst},\varepsilon_{\text{out}},\varepsilon_{\text{in}},\varepsilon_{a})\equiv F_{0}\left(\vec{2\theta },\vec{dst},\varepsilon_{\text{in}}+2\theta_{0},\varepsilon_{\text{out}}+2\theta_{0},\varepsilon_{0}+2\theta_{0},+\sqrt{\left|\varepsilon_{0}-\varepsilon_{a}\right|},+1\right)\\ F_{4}(\vec{dst},\varepsilon_{\text{out}},\varepsilon_{\text{in}},\varepsilon_{a})\equiv F_{0}\left(\vec{2\theta },\vec{dst},\varepsilon_{\text{in}}+2\theta_{0},\varepsilon_{\text{out}}+2\theta_{0},\varepsilon_{0}+2\theta_{0},-\sqrt{\left|\varepsilon_{0}-\varepsilon_{a}\right|},+1\right). \end{array}$$ (10)

Then, we create arrays for $I_{2}^{+}$ and $I_{2}^{-}$ which will be the accumulators as defined in caption of Table 1 of [5]. Also, we create an empty list which will accumulate index bounds. The semantics of this list will be that the + = operator concatenates the elements returned by the function onto the end. The algorithm is shown in algorithm 2.

#### 2.4.5 Computing *I*_3_ by Integrating *I*_2_

The previous two sections have presented the most complex part of the computation of the axial divergence peak shape. This section presents an implementation of Sec. 5 of [5], the computation of *I*_3_(*ε*, *β*) and the integral of it over all allowed *β* to get the total line shape. First, we create functions representing the transmission of Soller slits of angular full-width *β*_max_ for the incident Soller slit and angular full-width *γ*_max_ for the receiving Soller slit. These are from equation 24a, b of [5], slightly rewritten:

$$S_{i}(\beta )=\max(0,1-|2\beta /\beta_{\max}|)$$ (11)

$$S_{d}(\gamma )=\max(0,1-|2\gamma /\gamma_{\max}|).$$ (12)

Algorithm 3 shows how to carry out the integral over *β*.

**Algorithm 2:** Computation of *I*_2_

$\vec{I_{2}^{+}}=\text{array of zeros of same length as}\;\vec{\varepsilon }$
$\vec{I_{2}^{-}}=\text{array of zeros of same length as}\;\vec{\varepsilon }$
*indices*=[] # empty list
if *r_β_* is 1:
*indices*+= *F*_1_ ( $\vec{I_{2}^{+}}$, *ε_a_*, *ε*_0_, *ε_a_*, *ε_b_*)
*indices*+= *F*_2_ ( $\vec{I_{2}^{+}}$, *ε_b_*, *ε_a_*, *ε_b_*)
*indices*+= *F*_1_ ( $\vec{I_{2}^{-}}$, *ε_c_*, *ε*_0_, *ε_c_*, *ε_d_*)
*indices*+= *F*_2_ ( $\vec{I_{2}^{-}}$, *ε_d_*, *ε_c_*, *ε_d_*)
if *r_β_* is 2:
*indices*+= *F*_2_ ( $\vec{I_{2}^{+}}$, *ε_a_*, *ε*_0_, *ε_a_*)
*indices*+= *F*_3_ ( $\vec{I_{2}^{-}}$, *ε_b_*, *ε*_0_, *ε_a_*)
*indices*+= *F*_1_ ( $\vec{I_{2}^{-}}$, *ε_c_*, *ε_b_*, *ε_c_*, *ε_d_*)
*indices*+= *F*_2_ ( $\vec{I_{2}^{-}}$, *ε_d_*, *ε_c_*, *ε_d_*)
if *r_β_* is 3:
*indices*+= *F*_4_ ( $\vec{I_{2}^{-}}$, *ε_b_*, *ε_a_*, *ε_a_*)
*indices*+= *F*_1_ ( $\vec{I_{2}^{-}}$, *ε_c_*, *ε_b_*, *ε_c_*, *ε_d_*)
*indices*+= *F*_2_ ( $\vec{I_{2}^{-}}$, *ε_d_*, *ε_c_*, *ε_d_*)
*idxmin* = min(*indices*)
*idxmax* = max(*indices*)
returns $\vec{I_{2}^{+}}$, $\vec{I_{2}^{-}}$, *idxmin*, and *idxmax*

#### 2.4.6 Applying the Axial Divergence Convolution

To apply this convolution, which has been generated in real space, it must be transformed to Fourier space:

$$g(\vec{\omega })=\text{real\_fft}\left({\vec{I}}_{3}\right).$$ (13)

Because of the way the peak is centered, this transform has an alternating sign of −1 across its values. All odd-numbered bins of $g(\vec{\omega })$ will be multiplied by −1. Then, $\tilde{F}$ will be multiplied by $g(\vec{\omega })$.

**Algorithm 3:** Integration over *β* to get *I*_3_

${\vec{I}}_{3}=\text{array of zeros of same length as}\;\vec{\varepsilon }$
*β*_lim_ = min(*β*_2_, *β*_max_/2) # *β*_2_ from equation 4
# *nsteps* is the number of integration steps.
# values of 5–20 are usually sufficient.
for *j* between 0 and *nsteps:* # code below from equation 26a, b of [5]
*β* = *β*_lim_ × *j*/*nsteps*
$I_{2}^{+}$, $I_{2}^{-}$, *i*_0_, *i*_1_ computed according to section 2.4.4
*γ*_0_ = *β*/|cos 2*θ*|
$\vec{d\varepsilon }=\varepsilon_{0}-\vec{\varepsilon }[i_{0}:i_{1}]\#\vec{\varepsilon }$ is the $\vec{2\theta }-2\theta_{0}$ grid, from equation 5
$\vec{d\varepsilon }=\vec{d\varepsilon }\times 2\tan(2\theta_{0})$
$\vec{d\varepsilon }[0]=\max(0,\vec{d\varepsilon }[0])$
$\vec{d\varepsilon }[-1]=\max(0,\vec{d\varepsilon }[-1])$ # see note on array indexing in 2.1
$\vec{d\gamma }=\sqrt{\vec{d\varepsilon }}$
$\vec{\gamma^{+}}=\gamma_{0}+\vec{d\gamma }$
$\vec{\gamma^{-}}=\gamma_{0}-\vec{d\gamma }$
if *j* is 0 or j is last step: # trapezoidal rule endpoints
*weight* = 1
else:
*weight* = 2
$\vec{I_{3}}[i_{0}:i_{1}]=\vec{I_{3}}[i_{0}:i_{1}]+\left(\vec{I_{2}^{+}}[i_{0}:i_{1}]\times S_{d}\left(\vec{\gamma^{+}}\right)+\vec{I_{2}^{-}}[i_{0}:i_{1}]\times S_{d}\left(\vec{\gamma^{-}}\right)\times S_{i}[\beta ]\right)\times weight$
$\vec{I_{3}}=\vec{I_{3}}\times 2R^{2}|\tan2\theta_{0}|$
return $\vec{I_{3}}$ which is the full axial divergence function

### 2.5 Instrumental Effects (Other than Axial Divergence)

#### 2.5.1 Sample Offset and Zero Angle

The correction for the sample surface not lying in the equatorial plane of the diffractometer can be combined with the shift due to the zero angle error. If the sample is offset by *z*_0_, and the zero angle offset is 2*θ_z_*, the correction is

$$\delta_{z}=-2\frac{z_{0}\cos\theta }{R}+2\theta_{z}$$ (14)

and the convolver is:

$$g(\vec{\omega })=\exp(-i\vec{\omega }\delta_{z})$$ (15)

and $\tilde{F}$ is multiplied by $g(\vec{\omega })$

#### 2.5.2 Source Width and Tube Tails

The correction for the broad shoulders on the spatial distribution of emission for fine-focus X-ray tubes can be computed according to [7] and Sec. 4.1 of [8]. Define *w_m_* as the width of the central peak, *w_l_* as the distance to the low-angle side of the shoulder, *w_h_* as the distance to the high-angle side, and *I_t_* as the intensity of the tails, then,

$$\begin{array}{l} \varepsilon_{t}=\frac{w_{r}-w_{l}}{R}\\ \varepsilon_{m}=\frac{w_{m}}{R}\\ \delta_{t}=\frac{w_{r}+w_{l}}{R}\\ A_{r}=I_{t}\frac{w_{r}+w_{l}}{w_{m}} \end{array}$$ (16)

and the convolution is

$$g(\vec{\omega })=\mathrm{sinc}\left(\frac{\varepsilon_{m}\vec{\omega }}{2\pi }\right)+A_{t}\text{sinc}\left(\frac{\varepsilon_{t}\vec{\omega }}{2\pi }\right)\exp(-i\vec{\omega }\delta_{t})$$ (17)

and $\tilde{F}$ will be multiplied by this $g(\vec{\omega })$.

#### 2.5.3 Receiver Slit Equatorial Height

A receiver slit of full height *h_r_* creates a rectangular convolution of angular full width *h_r_*/*R* (where *R* is the diffractometer radius). From 42, the Fourier space representation is:

$$g(\vec{\omega })=\text{sinc}\frac{h_{r}\vec{\omega }}{2\pi R}$$ (18)

which will be multiplied into $\tilde{F}$.

#### 2.5.4 Flat Specimen/Equatorial Divergence Slit Size

From eqs. 9 and 10 of [8], the correction for the flat specimen error is

$$\varepsilon_{M}=\frac{\alpha^{2}}{2}\cot\theta_{0}$$ (19)

where *α* is the equatorial divergence angle of the X-ray beam. Then, the convolution function is

$$J_{FS}(\varepsilon )=\frac{1}{2\sqrt{-\varepsilon_{M}\varepsilon }}\text{where}-\varepsilon_{M}<\varepsilon <0$$ (20)

This is of the same form as *F*_0_, used in the axial divergence calculation (see Sec. 2.4.2), so we will use that function. Note that this is a computation in real space. This is shown in algorithm 4.

**Algorithm 4:** Computing the flat-specimen error

$\vec{J_{FS}}=\text{array of zeros of same length as}\vec{\;2\theta }\;\text{grid}$
*F*_0_ (
*ε*_0_ = 2*θ*_0_, $\vec{accum}=\vec{J_{FS}}$, *ε*_inner_ = 2*θ*_0,_ *ε*_outer_ = − *ε_M_*
$\vec{\varepsilon }=\vec{2\theta }$, $k=1/(2\sqrt{\varepsilon_{M}})$, *y*_0_ = 0
)
$g(\vec{\omega })=\text{real\_fft}\left(\vec{J_{FS}}\right)$ # transform real-space function to Fourier

Because of the way the peak is centered, this transform has an alternating sign of −1 across its values. All odd-numbered bins of $g(\vec{\omega })$ will be multiplied by −1. Then, $\tilde{F}$ will be multiplied by $g(\vec{\omega })$.

#### 2.5.5 Specimen Transparency

From equation 12 of [8], with a correction to *δ*, the convolution due to the finite interaction depth in the target is:

$$\begin{array}{l} J_{\mu }(\varepsilon )=\frac{\exp\frac{\varepsilon }{\delta }}{\delta \left(1-\exp\frac{\varepsilon_{\min}}{\delta }\right)}\;\text{where}\;\varepsilon_{\min}<\varepsilon <0\\ \varepsilon_{\min}=\frac{-2T\cos\theta_{0}}{R}\\ \delta =\frac{\sin2\theta_{0}}{2\mu R} \end{array}$$ (21)

where *μ* is the absorption coefficient of the sample, measured in units consistent with *R*, so if *R* is in mm, *μ* would be in mm^−1^, and *T* is the sample thickness in the same units as *R*. From 48, the Fourier transform of this expression is:

$$g(\vec{\omega })=\frac{1}{\delta }\frac{1-\exp(\varepsilon_{\min}(i\vec{\omega }+(1/\delta )))}{i\vec{\omega }+(1/\delta )}.$$ (22)

Then, $\tilde{F}$ will be multiplied by $g(\vec{\omega })$.

#### 2.5.6 Defocusing (Ω ≠ *θ*)

If the specimen angle Ω is offset from *θ*, a defocusing correction appears, per equation 15 of [8]. It is a rectangular convolution of angular full width

$$\delta_{DR}=\alpha \left(1-\frac{\sin(2\theta -\Omega )}{\sin\Omega }\right)$$ (23)

where *α* is the equatorial divergence angle. For small *θ* − Ω, this also reduces to:

$$\delta_{DR}\approx -\alpha (2\theta -2\Omega )\cot\theta .$$ (24)

From 42, the Fourier space representation is:

$$g(\vec{\omega })=\text{sinc}\frac{\delta_{DR}\vec{\omega }}{2\pi }$$ (25)

which will be multiplied into $\tilde{F}$.

#### 2.5.7 Silicon Position Sensitive Detector (Si PSD)

A Si PSD, which has a finite window width, suffers defocusing due to two effects. The first is the effect discussed above, due to the sample angle Ω being different from the diffraction angle *θ*, resulting in a violation of the expected parafocusing optics. The second effect is due to the flat face of the detector itself; active pixels are not located on the radius defined by the diffractometer configuration. Unlike corrections for older PSDs [15], which include parallax due to the long absorption length in a gas-filled detector, we assume the detector is planar and has no effective depth.

The exact expression for a ray starting at the source at an angle *α* from the center ray, being diffracted by an angle 2*θ*, and striking the detector face which is centered at 2*θ* + *ε*, is a ray which intersects the detector face at a position *y*:

$$y=-\frac{1}{2}R\frac{\cos(2\alpha -\Omega -\varepsilon )-2\sin(\alpha )\sin(\alpha -\Omega +2\theta )-\cos(\varepsilon -\Omega )}{\sin(\alpha -\Omega )\cos(\alpha -\varepsilon )}.$$ (26)

This can be expanded as a series in *ε* and *α*, which is:

$$y=-R\varepsilon +\alpha R\left(\left(1-\frac{\sin(2\theta -\Omega )}{\sin\Omega }\right)+\varepsilon^{2}\left(1-\frac{1}{2}\frac{\sin(2\theta -\Omega )}{\sin\Omega }\right)\right)+O(\varepsilon^{3})+O(\alpha^{2}).$$ (27)

The first term is just the expected offset of the peak on the detector face, and is not an aberration. The second term contains two components, the first of which is exactly that of 23. The second component depends on *ε*^2^. In the limit that *θ* → Ω, it reduces to simply *ε*^2^/2. Thus,

$$\delta_{\text{psd}}\equiv \frac{y}{R}\approx \alpha \left(1-\frac{\sin(2\theta -\Omega )}{\sin\Omega }\right)+\alpha \frac{\varepsilon^{2}}{2}$$ (28)

or, using the simplification of 24,

$$\delta_{\text{psd}}\approx -2\alpha \varepsilon \cot\theta +\alpha \frac{\varepsilon^{2}}{2}.$$ (29)

Now, we need to consider the relative magnitudes of these two terms. For a detector which is 1 cm in length, and 20 cm from the sample, *ε* = 0.05, so *ε*^2^ = 0.0025, and the quadratic is much smaller than the linear one for almost all *θ*. At high angles, where cot*θ* suppresses the first term, the aberration is still typically very small, since *α* may be of order 1° and *αε*^2^/2 = 0.00125° which is a very small contribution to the width for a high-angle peak. We present below an exact solution to the case in which the *ε*^2^ is ignored. In the case it is needed, the integral below needs to be carried out numerically.

If the detector window being analyzed extends from distance *y*_1_ to distance *y*_2_ above the centerline, and is centered so that *θ*_0_ = Ω,

$$g(\vec{\omega })=\int_{y_{1}/R}^{y_{2}/R}\text{sinc}\frac{\delta_{\text{psd}}\vec{\omega }}{2\pi }d2\theta^{\prime }$$ (30)

where *δ*_psd_ comes from 29. Note that the defocusing correction of Sec. 2.5.6 is symmetrical in 2*θ*, so it is only necessary to integrate over one side of the detector.

The integral can be carried out analytically using the approximation of 24, resulting in an expression involving the sine integral function Si(*x*), where

$$\text{Si}(x)=\int_{0}^{x}\frac{\sin u}{u}du$$ (31)

$$\begin{array}{l} g(\vec{\omega })=\int_{y_{1}/R}^{y_{2}/R}\text{sinc}\frac{(\alpha (2\theta^{\prime })\cot\theta_{0})\vec{\omega }}{2\pi }d2\theta^{\prime }\\ g(\vec{\omega })=\frac{2}{\alpha \cot\theta_{0}\vec{\omega }}\left(\text{Si}\frac{\alpha y_{2}\cot\theta_{0}\vec{\omega }}{2R}-\text{Si}\frac{\alpha y_{1}\cot\theta_{0}\vec{\omega }}{2R}\right). \end{array}$$ (32)

Note that evaluation of this function involves a 0/0 where *ω* = 0. However, the limit can easily be taken analytically. The zero bin of $g(\vec{\omega })$ should be set to (*y*_2_ − *y*_1_)/*R*, which is just the angular length of the exposed detector face. Also note that the subtracted term in 32 vanishes if *y*_1_ = 0.

For a symmetrical detector, *y*_1_ = 0. However, some data analyses may split the data from the detector into a pattern using the central pixels of the detector to get a high-resolution result, and then use the remaining pixels to create another pattern which has lower angular resolution, but takes advantage of the counting statistics available over the full detector aperture. In this case, the ‘central’ pattern would use *y*_1_ = 0 and *y*_2_ = *W_hr_* where *W_hr_* is the half-height of the region to be sampled for high-resolution data. The ‘outer’ pattern would use *y*_1_ = *W_hr_* and *y*_2_ = *W_det_* where *W_det_* is the half-height of the full detector aperture. Such a split would permit nearly optimal use of the characteristics of the PSD, with high resolution and high active area.

The form of 32 gives direct guidance as to where the cutoff *W_hr_* should be made. For small arguments, Si(*ax*)/*a* ≈ *x* − *a*^2^ *x*^3^/18 = *x*(1− *a*^2^*x*^2^/18). Thus, if *y*_1_ = 0,

$$g(\omega )\approx \frac{y_{2}}{R}\left(1-\frac{1}{18}{\left(\frac{\alpha y_{2}\cot\theta_{0}\omega }{2R}\right)}^{2}\right).$$ (33)

If the second term is small, the convolver does not roll off at high frequencies. By examining the other terms in the Fourier transform $\tilde{F}$ one can find a frequency *ω_max_* at which other terms dominate and have rolled off most of the response. Then, if

$$W_{hr}\underset{˜}{<}\frac{6\sqrt{2}R}{\alpha \cot\theta_{0}\omega_{max}}$$ (34)

the Si PSD will not significantly broaden the overall response of the system as a result of this window selection. A more advanced approach would be to adjust this window width as a function of position in 2*θ*, so that as the peaks are dispersion-broadened at high angle, one would automatically use a wider window to collect more counts with no loss of resolution.

As with all the other corrections, $\tilde{F}$ will be multiplied by the resulting $g(\vec{\omega })$.

##### Aside on real-space solution

If one is working in real space, rather than Fourier space, the integral of a top-hat function over widths *δ_DR_* of 24 can be carried out analytically to get the convolution function. As before, assuming the window extends from *y*_1_ to *y*_2,_, the result is:

$$f(2\theta -2\theta_{0})\equiv f(\varepsilon )=\{\begin{array}{ll} 0 & |\varepsilon |<\frac{y_{1}\alpha \cot\theta_{0}}{R}\\ -\log\frac{R|\varepsilon |}{y_{2}\alpha \cot\theta_{0}} & \frac{y_{1}\alpha \cot\theta_{0}}{R}<|\varepsilon |<\frac{y_{2}\alpha \cot\theta_{0}}{R}\\ 0 & |\varepsilon |>\frac{y_{2}\alpha \cot\theta_{0}}{R} \end{array}.$$ (35)

This expression includes a weak singularity at *ε* = 0 (if *y*_1_ = 0, which is the most likely case), which can be handled in much the same manner as the $1/\sqrt{x}$ singularity which was handled by the helper function in Sec. 2.4.2. Instead of integrating $1/\sqrt{x}\to 2\sqrt{x}$, one integrates the log to get

$$-\int_{0}^{\varepsilon }\log\frac{R\varepsilon^{\prime }}{y_{2}\alpha \cot\theta_{0}}d\varepsilon^{\prime }=\varepsilon \left(1-\log\frac{R\varepsilon }{y_{2}\alpha \cot\theta_{0}}\right).$$ (36)

This can then have the forward difference computed, as in Sec. 2.4.2, to get the appropriate singularity-free binned function. A full code example is not provided, since it is essentially identical to the helper code.

### 2.6 Conversion of Transform Results to 2*θ* Space

In the previous sections, we have enumerated many convolutions that get accumulated multiplicatively into $\tilde{F}$. Any other aberrations which are to be included can be done so in a similar manner. When everything is included, one needs to un-transform $\tilde{F}$ into real space to get $F(\vec{2\theta }-2\theta_{0})$, the nearly-final aberration function. We have been assuming the user has a Fourier transform library which provides a pair of properly-matched transform functions. $\text{real\_fft}(\vec{x})$ takes a real array $\vec{x}$ of length *N* and transforms it into the positive *ω* components of the Fourier transform, a complex array of length *N*/2 + 1. We apply its inverse, to get

$$F(\vec{2\theta }-2\theta_{0})=\text{inverse\_real\_fft}(\tilde{F})$$ (37)

which takes *N*/2 + 1 complex numbers and converts them back to *N* real elements.

The one drawback to working in Fourier space is that all functions have built-in an implied periodicity of the length over which they are sampled. For many functions, this is not an issue, since they go quickly to zero near their boundaries, and so no wrap-around occurs. Unfortunately for the situation here, the aberration functions have extremely long Lorentzian tails, due to the contributions from the *h_k_* from 1. These tails always wrap around, which results in their inaccurate computation unless one spends much extra computational effort by computing the function over a very large interval in 2*θ* space.

**Fig. 2:**
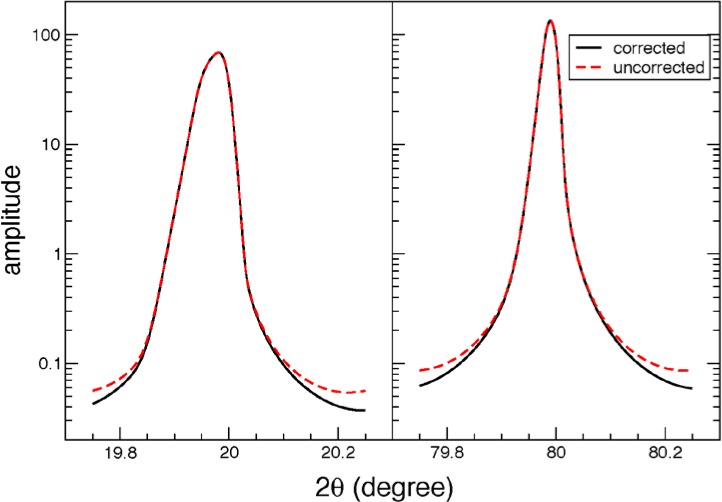
Correction due to periodic Fourier transform, shown at low angle where the peak is very asymmetrical, and at mid-angle where it is nearly symmetrical. Note that for the left-hand case, the 2*θ* window is barely wide enough, so the peak tails are still very asymmetrical.

This problem has been addressed previously in a paper on the computation of Voigt functions by Fourier transform methods [16]. In short, the periodicity is equivalent to having computed the transform of an infinitely repeating comb of the line shape. Since the long tail is almost perfectly Lorentzian, one can subtract the infinite sum of Lorentzians from the computed aberration, which corrects the tail. This can be done in closed form. From equation 7 of [16], the sum is:

$$\varepsilon (\vec{2\theta }-\mu )=\frac{\sinh\frac{2\pi \alpha }{\Delta }}{\Delta \left(\cosh\frac{2\pi \alpha }{\Delta }-\cos\frac{2\pi (\vec{2\theta }-\mu )}{\Delta }\right)}-\left(\frac{\alpha }{\pi }\right)\frac{1}{{\left(\vec{2\theta }-\mu \right)}^{2}+\alpha^{2}}$$ (38)

where Δ is the full width of the 2*θ* window, *μ* is the computed centroid of the peak, and *α* is the half-width of the widest component of the Lorentzian. This function is normalized to unit area. Then, the corrected shape is:

$$F_{c}(\vec{2\theta }-2\theta_{0})=F(\vec{2\theta }-2\theta_{0})-A\varepsilon (\vec{2\theta }-\mu )$$ (39)

where *A* is the area of $F(\vec{2\theta })$. This makes a very good correction of the tails, assuming that the peak is not so asymmetrical that it has quite different amplitudes at the boundaries of the 2*θ* window. An example of the correction is shown in Fig. 2.

## 3. Numerical Comparisons

The data in Secs. 3.1 and 3.2 are provided to allow one to compare implementations of the NIST FPAPC to the results from Topas. Section 3.3, with comparisons to data, is provided as general validation of the FPA for some important test cases. While the FPAPC is not a Rietveld code, in that it does not utilize a full structural mode, it can utilize space group symmetry (of various SRM materials) to constrain peak positions to a single lattice parameter, equivalent to a Pawley fit.

### 3.1 Simplified Source Spectrum, Variable Soller Slits

This section presents numerical and graphical comparisons of the output of FPAPC with that of Topas. The setup we are comparing is that of a diffractometer with the parameters shown in Table 2, using the point detector with the “full” model for axial divergence. These examples have the source spectrum artificially restricted, to make the effect of axial divergence and Soller slits more evident.

**Fig. 3:**
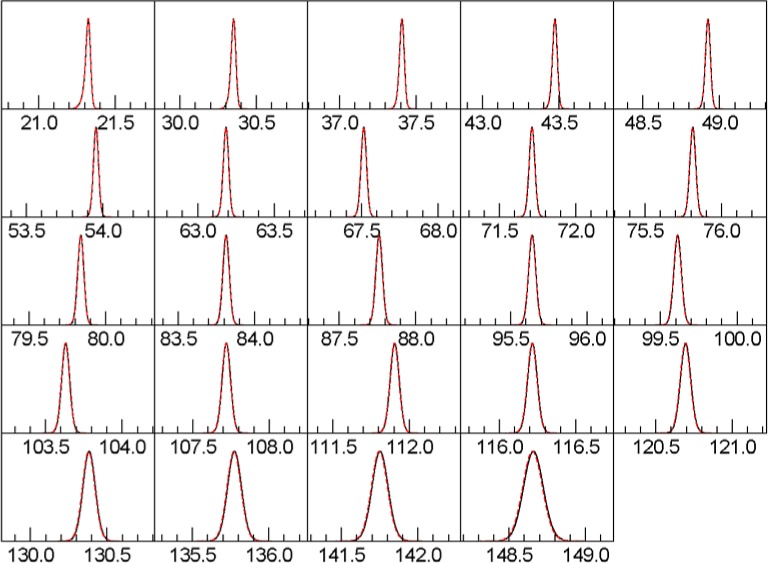
Line shapes with 2.5° Soller slits. Red, dotted curve is Topas. Black curve is this work.

**Fig. 4:**
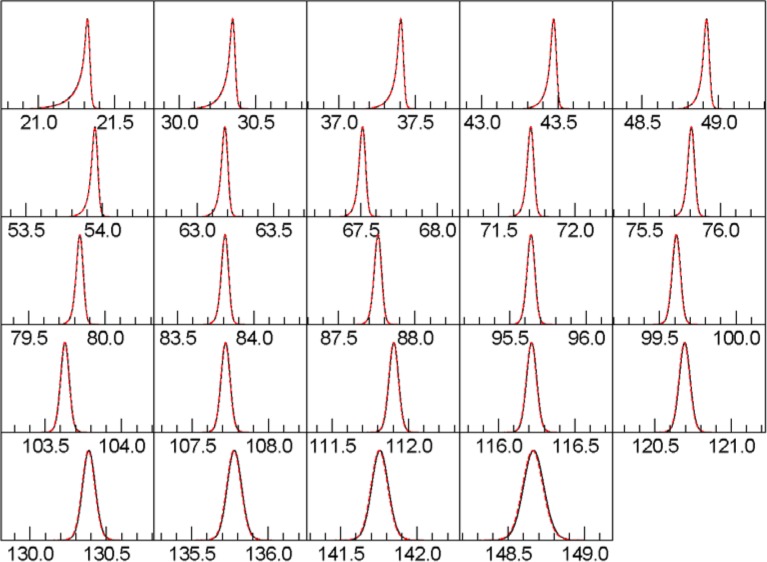
Line shapes with 5.3° Soller slits. Red, dotted curve is Topas. Black curve is this work.

**Fig. 5:**
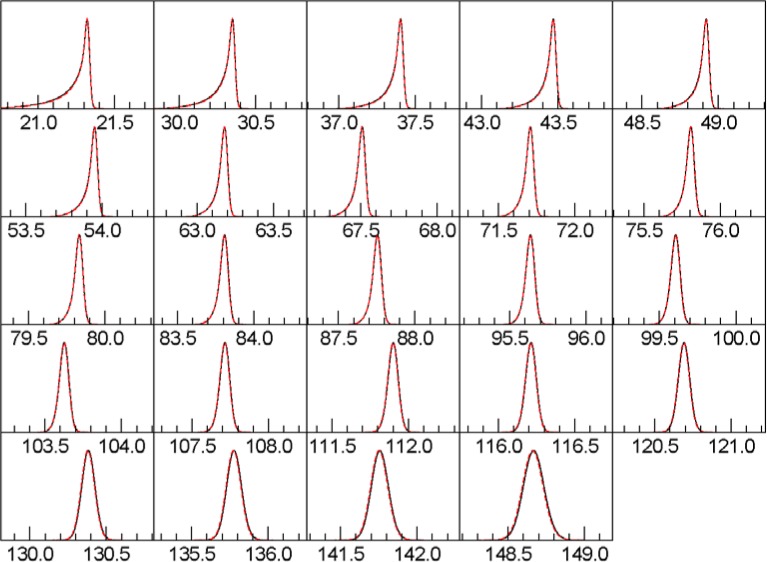
Line shapes with 10.6° Soller slits. Red, dotted curve is Topas. Black curve is this work.

We compute synthetic peak patterns for material with the characteristics of SRM 660c LaB_6_. Each data set is computed with different Soller slit settings, ranging from 2.5° full-width to 10.6° full-width. Tables 3, 4 and 5 show the detailed errors, and Figs. 3, 4, and 5 show the peak shapes for the 2.5°, 5.3°, and 10.6° cases, respectively.

#### Column Descriptions

(h,k,l) the reflection for this peak
tp top the angle of the highest point in the peak from Topas
py top same for the python implementation of this algorithm
Δ_1_ (tp top)-(py top)
tp ζ the distance between the centroid and the top, a measure of asymmetry
py ζ same for the python implementation of this algorithm
Δ_2_ (tp ζ)-(py ζ)
tp IB integral breadth of the peak from Topas
py IB same for the python implementation of this algorithm
% err fractional error in the IB

### 3.2 Realistic Source Spectrum and Parameters

**Fig. 6:**
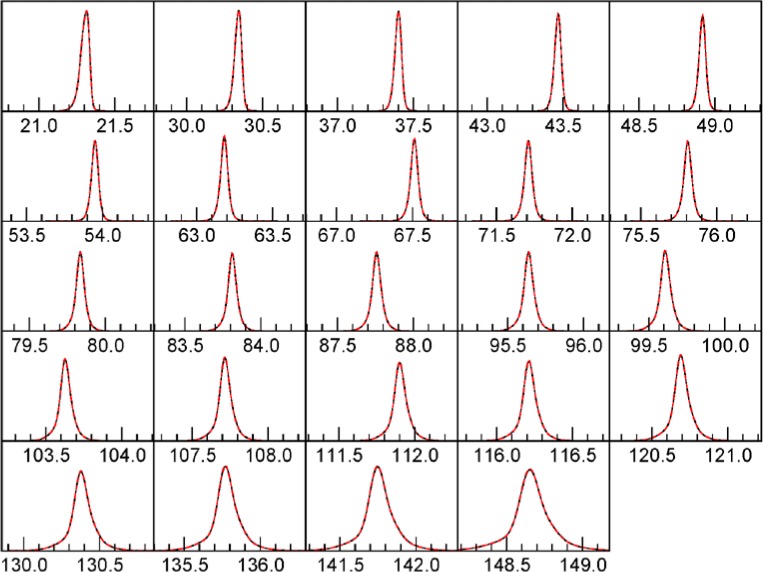
Line shapes with full Rietveld. Red, dotted curve is Topas. Black curve is this work.

We now compute a sample with a more realistic, full spectrum as determined from fits to data from the NIST Johansson incident beam monochromator, as described in [1]. All parameters shown in Table 6 were fit by Topas and FPAPC. Table 7 shows the detailed errors, and Fig. 6 shows the peak shapes.

### 3.3 Comparison to Measurements

**Fig. 7:**
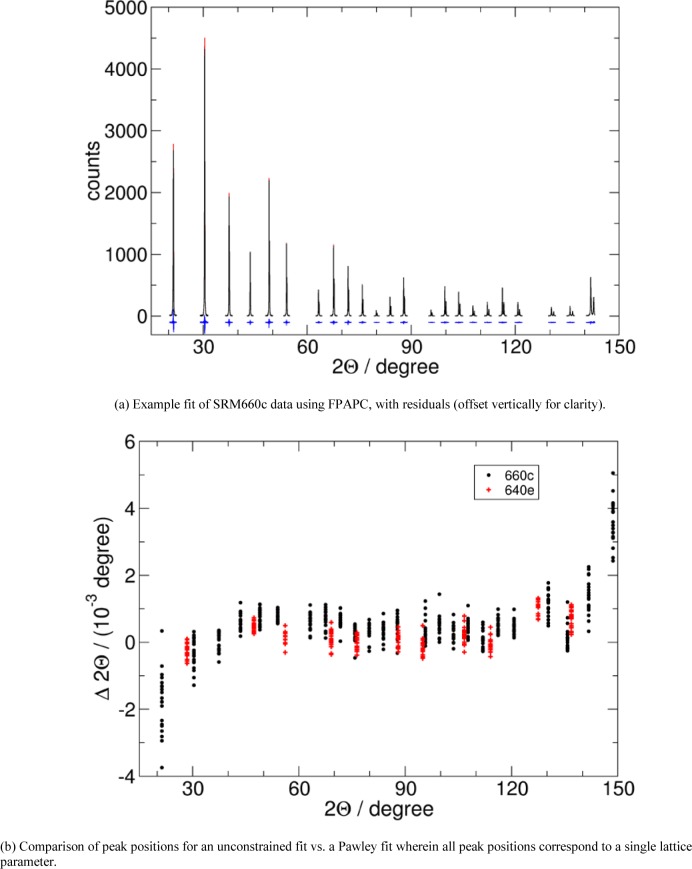
Example fit, and peak position errors for SRMs 640e and 660c.

The most critical metric for comparison of the two programs is that of refined lattice parameter. SRMs 640e [17] and 660c [18] were certified in March, 2015 using data from the instrument described in [1]. The certification procedure involved the collection of twenty high-quality (24 hour scans) data sets for each of the two SRMs. These were analyzed independently using Topas with a Pawley analysis. With the NIST FPAPC, the twenty data sets were analyzed with a single, global refinement: specimen specific parameters, such as specimen displacement, were refined independently while instrument specific parameters, common across all data sets, were refined as single parameters. In Fig. 7 we show a typical fit to the data. Close correspondence between the instrument specific parameters obtained from Topas and those from FPAPC was observed. Additional testing indicated the residual error terms were not increased significantly by the variation of the instrument specific terms within the “window” of refined values obtained with the two codes. Instrument parameters, common to all data sets were then fixed at values that largely constituted the average values obtained with the two codes. This being done, the average of the lattice parameters values obtained from the average of the 20 independent Topas analyses, for both SRMs 640e and 660c, agreed with the corresponding global FPAPC values to within 2 fm.

Testing of the FPA model itself can be performed with an analysis of the variation in lattice parameter with reflection position in 2*θ* as reported previously [19]. This is a very sensitive test of the success of the FPA model as all the reflections in a pattern should give the same lattice parameter. Lattice parameter is the only property that is absolutely conserved across the entire pattern while profile asymmetry can vary dramatically in both degree and direction with 2*θ*. Again we used SRMs 640e and 660c for this purpose. In Fig. 7 we show the comparison of the peak positions from a globally refined lattice parameter, determined with FPAPC, with peak positions when refined independently. For a wide range of angles, from roughly 40° to 140° in 2*θ*, the corrections provided by the FPA are very good. There is, however, a clear, systematic tendency at low and high angles, where the peaks are most asymmetric, for the result to be biased. This is not understood, and is a matter of intense focus by the authors at this time. It is worth noting that the information of highest quality about the lattice parameter of the material comes from the peaks in the 50°–120° 2*θ* range, where contribution of aberrations are minimal and the angle is high enough that the contribution of a small angular error to the lattice parameter is minimized.

**Fig. 8:**
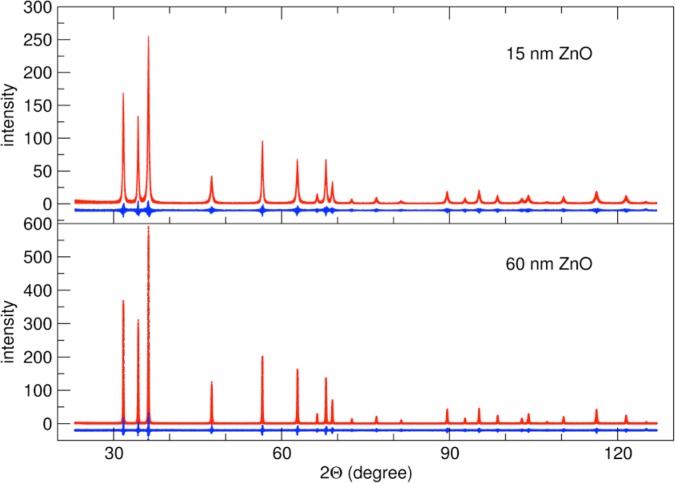
Full-pattern FPAPC fit to patterns from SRM1979-type ZnO 15 nm and 60 nm particles.

SRM 1979 is being certified for the measurement of crystallite size from an analysis of profile broadening. It was prepared by decomposing zinc oxalate in a large-scale, NIST-built vacuum furnace using a heating schedule derived from the procedures outlined in [20, 21]. The ZnO was then annealed in air to obtain two powders, one with an approximate crystallite size of 15 nm and a second one of 60 nm. In Fig. 8, we show a result of applying FPAPC code, extended to carry out the Scardi and Leoni model for log-normal crystallite size distributions [22] and the stacking fault density model of Warren [23]. This demonstrates that the algorithms described above can be extended to include complex models for material microstructure, many of which have natural representations in Fourier space. The breadth of the peaks in diffraction patterns from these ZnO materials varies widely due to the both crystallite size and the hkl specific stacking-faults.

## 4. Discussion

The Fundamental Parameters Approach to X-ray powder diffraction line profile analysis has played a central role in NIST powder diffraction SRM development since its inception with the aforementioned work of Cheary, Coelho and Cline (and collaborators on various SRM projects). We have demonstrated that the refined lattice parameter values obtained with our independently written NIST FPAPC and those of the commercial code Topas, that NIST has used since the year 2000 for SRM certification, agree to within 2 fm. This observation would confirm that both the NIST FPAPC and Topas are preforming in accordance to published FPA models. This conclusion is further supported by the data presented in Secs. 3.1 and 3.2 that illustrate that the form of the FPA profiles from the two programs are essentially identical. The equivalence of results between the NIST open implementation of FPA models and those from Topas enhances the transparency of the analyses performed in the certification of NIST SRMs for powder diffraction.

## Figures and Tables

**Table 1:** Selection of computation boundaries and *β* ranges

Condition 1	and		Condition 2	
*r_β_* ↓ \*ε* →	*ε_a_*	*ε_b_*	*ε_c_*	*ε_d_*
$L_{r}>Z_{0}^{+}-Z_{0}^{-}$		$Z_{0}^{+}\leq \frac{L_{r}}{2}$ and $Z_{0}^{-}\geq -\frac{L_{r}}{2}$		
1	$\varepsilon_{1}^{+}$	$\varepsilon_{2}^{+}$	$\varepsilon_{1}^{-}$	$\varepsilon_{2}^{-}$
		$\left(Z_{0}^{+}>\frac{L_{r}}{2}\text{and}\;Z_{0}^{-}<\frac{L_{r}}{2}\right)$ or $Z_{0}^{+}>-\frac{L_{r}}{2}\text{and}\;Z_{0}^{-}<-\frac{L_{r}}{2}$		
2	$\varepsilon_{2}^{+}$	$\varepsilon_{1}^{+}$	$\varepsilon_{1}^{-}$	$\varepsilon_{2}^{-}$
		any other range of *Z*_0_		
3	$\varepsilon_{2}^{+}$	$\varepsilon_{1}^{+}$	$\varepsilon_{1}^{-}$	$\varepsilon_{2}^{-}$
$L_{r}<Z_{0}^{+}-Z_{0}^{-}$		$Z_{0}^{+}\geq \frac{L_{r}}{2}$ and $Z_{0}^{-}\leq -\frac{L_{r}}{2}$		
1	$\varepsilon_{1}^{-}$	$\varepsilon_{2}^{+}$	$\varepsilon_{1}^{+}$	$\varepsilon_{2}^{-}$
		$\left(Z_{0}^{+}>\frac{L_{r}}{2}\text{and}-\frac{L_{r}}{2}<Z_{0}^{-}<\frac{L_{r}}{2}\right)$ or $\left(-\frac{L_{r}}{2}<Z_{0}^{+}<\frac{L_{r}}{2}\;\text{and}\;Z_{0}^{-}<-\frac{L_{r}}{2}\right)$		
2	$\varepsilon_{2}^{+}$	$\varepsilon_{1}^{-}$	$\varepsilon_{1}^{+}$	$\varepsilon_{2}^{-}$
		any other range of *Z*_0_		
3	$\varepsilon_{2}^{+}$	$\varepsilon_{1}^{-}$	$\varepsilon_{1}^{+}$	$\varepsilon_{2}^{-}$

**Table 2:** Topas model parameters

Parameter	Value	Parameter	Value
Zero error	−0.026°	Displacement	−0.011 mm
*R_p_*, *R_s_*	217.5 mm	Rec. slit width	75 *µ*m
Fil. length	15 mm	Samp. length	15 mm
Rec. slit length	5	Sample absorption	137.4 cm^−1^
*CS_L_*	3134 nm	*CS_G_*	379 nm
Lattice spacing	4.15695 Å		
**Source Spectrum**			
intensity (*l_a_*)	wavelength (Å) (*l_o_*)	Lor. width (mÅ) (*l_h_*)	Gauss. width (mÅ) (*l_g_*)
1	1.540591	0	0.4323

**Table 3:** 2.5° full width Soller slits

(h,k,l)	tp top (°)	py top (°)	Δ_1_ (m°)	tp ζ (m°)	py ζ (m°)	Δ_2_ (m°)	tp IB (m°)	py IB (m°)	% err
(0, 0, 1)	21.3224	21.3226	−0.12	−4.9	−5.1	0.23	47	46	0.78
(0, 1, 1)	30.3499	30.3500	−0.12	−2.3	−2.4	0.18	43	44	−0.42
(1, 1, 1)	37.4072	37.4074	−0.17	−1.3	−1.5	0.14	44	43	0.44
(0, 0, 2)	43.4723	43.4723	−0.01	−1.0	−1.0	0.01	42	44	−1.49
(0, 1, 2)	48.9233	48.9237	−0.32	−0.6	−0.8	0.18	45	45	0.07
(1, 1, 2)	53.9551	53.9551	0.03	−0.7	−0.6	−0.12	45	45	−0.01
(0, 2, 2)	63.1849	63.1850	−0.08	−0.5	−0.4	−0.12	47	48	−1.02
(0, 0, 3)	67.5141	67.5143	−0.29	−0.2	−0.3	0.13	49	49	−0.07
(0, 1, 3)	71.7118	71.7122	−0.34	0.1	−0.2	0.31	51	51	0.20
(1, 1, 3)	75.8111	75.8110	0.16	−0.5	−0.2	−0.34	52	52	0.01
(2, 2, 2)	79.8366	79.8369	−0.28	−0.1	−0.3	0.20	53	54	−1.33
(0, 2, 3)	83.8126	83.8125	0.07	−0.2	−0.1	−0.07	55	57	−0.97
(1, 2, 3)	87.7589	87.7590	−0.05	−0.2	−0.2	0.02	58	59	−0.83
(0, 0, 4)	95.6387	95.6387	−0.07	0.1	−0.1	0.17	62	64	−1.11
(0, 1, 4)	99.6100	99.6099	0.09	0.1	−0.1	0.12	66	67	−0.71
(1, 1, 4)	103.6287	103.6289	−0.18	0.1	0.0	0.09	70	70	−0.10
(1, 3, 3)	107.7175	107.7175	0.05	−0.1	−0.1	−0.01	73	74	−1.01
(0, 2, 4)	111.9019	111.9020	−0.07	−0.0	−0.0	−0.03	79	79	0.13
(1, 2, 4)	116.2138	116.2136	0.20	−0.2	0.1	−0.26	82	84	−0.92
(2, 3, 3)	120.6918	120.6919	−0.05	0.1	0.1	−0.03	89	90	−0.92
(2, 2, 4)	130.3790	130.3791	−0.10	0.1	0.0	0.10	106	108	−0.96
(0, 0, 5)	135.7712	135.7715	−0.28	0.2	−0.0	0.19	121	121	0.16
(1, 3, 4)	141.7472	141.7474	−0.15	0.3	0.2	0.12	140	140	0.14
(3, 3, 3)	148.6525	148.6527	−0.23	0.2	0.1	0.11	168	171	−0.99

**Table 4:** 5.3° full width Soller slits

(h,k,l)	tp top (°)	py top (°)	Δ_1_ (m°)	tp ζ (m°)	py ζ (m°)	Δ_2_ (m°)	tp IB (m°)	py IB (m°)	% err
(0, 0, 1)	21.3179	21.3175	0.41	−38.8	−39.9	1.04	79	81	−1.02
(0, 1, 1)	30.3451	30.3448	0.24	−23.8	−24.6	0.72	69	71	−1.69
(1, 1, 1)	37.4020	37.4022	−0.20	−17.1	−18.1	1.01	65	66	−0.18
(0, 0, 2)	43.4674	43.4671	0.27	−13.5	−13.9	0.37	62	63	−0.94
(0, 1, 2)	48.9182	48.9184	−0.19	−10.6	−11.2	0.63	62	62	−0.15
(1, 1, 2)	53.9500	53.9501	−0.14	−8.9	−9.3	0.40	61	61	−0.24
(0, 2, 2)	63.1801	63.1802	−0.03	−6.3	−6.4	0.16	59	61	−1.13
(0, 0, 3)	67.5098	67.5097	0.14	−5.4	−5.4	−0.02	61	61	−0.13
(0, 1, 3)	71.7077	71.7079	−0.21	−4.4	−4.6	0.24	61	62	−0.32
(1, 1, 3)	75.8064	75.8066	−0.19	−3.5	−3.8	0.31	62	62	−0.38
(2, 2, 2)	79.8324	79.8325	−0.13	−2.8	−3.0	0.26	63	64	−1.08
(0, 2, 3)	83.8090	83.8088	0.13	−2.7	−2.5	−0.19	63	65	−1.74
(1, 2, 3)	87.7552	87.7552	0.03	−1.9	−2.0	0.09	64	67	−1.57
(0, 0, 4)	95.6358	95.6355	0.23	−1.2	−1.3	0.06	69	70	−1.10
(0, 1, 4)	99.6073	99.6070	0.36	−0.9	−0.9	−0.01	71	73	−1.18
(1, 1, 4)	103.6264	103.6264	0.01	−0.5	−0.7	0.13	75	76	−0.30
(1, 3, 3)	107.7156	107.7153	0.24	−0.5	−0.5	0.03	77	79	−1.09
(0, 2, 4)	111.9004	111.9002	0.23	−0.1	−0.2	0.02	83	83	−0.03
(1, 2, 4)	116.2127	116.2125	0.20	−0.1	−0.0	−0.06	87	88	−0.82
(2, 3, 3)	120.6916	120.6915	0.08	0.1	0.1	−0.03	92	94	−1.23
(2, 2, 4)	130.3804	130.3801	0.25	0.2	0.4	−0.24	110	112	−0.96
(0, 0, 5)	135.7737	135.7731	0.57	0.2	0.5	−0.27	125	125	0.09
(1, 3, 4)	141.7508	141.7504	0.38	0.6	0.8	−0.18	144	144	−0.01
(3, 3, 3)	148.6577	148.6577	−0.05	0.9	0.8	0.11	173	176	−0.88

**Table 5:** 10.6° full width Soller slits

(h,k,l)	tp top (°)	py top (°)	Δ_1_ (m°)	tp ζ (m°)	py ζ (m°)	Δ_2_ (m°)	tp IB (m°)	py IB (m°)	% err
(0, 0, 1)	21.3160	21.3157	0.25	−88.4	−89.9	1.57	114	118	−1.76
(0, 1, 1)	30.3429	30.3423	0.59	−57.2	−58.2	0.93	96	101	−2.72
(1, 1, 1)	37.3998	37.3998	0.09	−43.3	−44.2	0.89	90	92	−0.73
(0, 0, 2)	43.4640	43.4638	0.21	−34.1	−34.9	0.73	84	88	−2.51
(0, 1, 2)	48.9154	48.9150	0.40	−28.7	−29.0	0.27	82	85	−1.43
(1, 1, 2)	53.9465	53.9465	0.07	−24.0	−24.6	0.60	80	82	−1.29
(0, 2, 2)	63.1762	63.1763	−0.08	−17.6	−18.0	0.44	76	78	−1.76
(0, 0, 3)	67.5059	67.5057	0.17	−15.4	−15.6	0.20	76	78	−0.81
(0, 1, 3)	71.7038	71.7038	−0.02	−13.2	−13.6	0.32	76	77	−0.58
(1, 1, 3)	75.8026	75.8028	−0.22	−11.4	−11.7	0.31	77	77	0.31
(2, 2, 2)	79.8290	79.8287	0.23	−10.2	−10.1	−0.05	74	77	−1.65
(0, 2, 3)	83.8049	83.8050	−0.09	−8.6	−8.8	0.14	75	78	−1.83
(1, 2, 3)	87.7512	87.7516	−0.44	−7.1	−7.4	0.31	76	78	−1.17
(0, 0, 4)	95.6317	95.6312	0.52	−5.0	−5.1	0.18	78	81	−1.47
(0, 1, 4)	99.6038	99.6030	0.74	−4.5	−4.2	−0.21	80	82	−1.36
(1, 1, 4)	103.6229	103.6222	0.70	−3.5	−3.4	−0.18	83	84	−0.40
(1, 3, 3)	107.7121	107.7115	0.65	−2.9	−2.6	−0.28	84	86	−1.23
(0, 2, 4)	111.8971	111.8966	0.44	−2.1	−2.0	−0.09	90	90	−0.01
(1, 2, 4)	116.2095	116.2091	0.39	−1.5	−1.5	−0.05	92	94	−0.96
(2, 3, 3)	120.6887	120.6882	0.49	−1.0	−0.8	−0.21	97	99	−1.21
(2, 2, 4)	130.3782	130.3779	0.32	−0.1	−0.0	−0.10	113	115	−0.90
(0, 0, 5)	135.7720	135.7715	0.49	0.1	0.5	−0.31	128	128	0.04
(1, 3, 4)	141.7499	141.7497	0.12	0.8	0.7	0.12	147	147	0.02
(3, 3, 3)	148.6580	148.6579	0.07	1.1	1.0	0.11	175	179	−0.87

**Table 6:** Topas full Rietveld model parameters

Parameter	Value	Parameter	Value
Zero Error	−0.0268°	Displacement	−0.016 mm
*R_p_*, *R_s_*	217.5 mm	Rec. slit width	75 *µ*m
Fil. Length	8 mm	Samp. Length	15 mm
Rec. slit length	12	Sample Absorption	126.8 cm^−1^
*CS_L_*	3027 nm	*CS_G_*	488 nm
Lattice spacing	4.156925692 Å	Equat. Diverg.	1.096°
**Source Spectrum**			
intensity (*l_a_*)	wavelength (Å) (*l_o_*)	Lor. width (mÅ) (*l_h_*)	Gauss. width (mÅ) (*l_g_*)
1	1.540591	0	0.4323
0.7504	1.540591	0	1.6718
0.0418	1.540591	0	3.9651
0.1861	1.541064	0	0.4565

**Table 7:** Comparison of pattern with full Rietveld fit from Topas

(h,k,l)	tp top (°)	py top (°)	Δ_1_ (m°)	tp ζ (m°)	py ζ (m°)	Δ_2_ (m°)	tp IB (m°)	py IB (m°)	% err
(0, 0, 1)	21.3078	21.3085	−0.72	−8.0	−8.1	0.03	78	76	1.22
(0, 1, 1)	30.3409	30.3410	−0.14	−4.5	−4.2	−0.27	61	62	−0.48
(1, 1, 1)	37.4001	37.4006	−0.53	−2.7	−2.9	0.17	62	59	1.75
(0, 0, 2)	43.4665	43.4671	−0.57	−2.0	−2.2	0.22	59	60	−0.17
(0, 1, 2)	48.9187	48.9191	−0.33	−1.8	−1.9	0.03	62	61	0.74
(1, 1, 2)	53.9511	53.9513	−0.16	−1.8	−1.6	−0.14	63	62	0.67
(0, 2, 2)	63.1822	63.1820	0.15	−1.7	−1.4	−0.30	66	67	−0.31
(0, 0, 3)	67.5121	67.5121	0.00	−1.6	−1.3	−0.32	70	69	0.64
(0, 1, 3)	71.7100	71.7105	−0.43	−1.2	−1.2	−0.05	73	72	0.40
(1, 1, 3)	75.8092	75.8094	−0.16	−1.3	−1.1	−0.24	76	75	0.69
(2, 2, 2)	79.8353	79.8355	−0.21	−1.2	−0.9	−0.26	78	78	−0.05
(0, 2, 3)	83.8110	83.8116	−0.57	−0.6	−0.7	0.13	81	81	−0.30
(1, 2, 3)	87.7580	87.7581	−0.05	−0.9	−0.6	−0.29	84	85	−0.57
(0, 0, 4)	95.6384	95.6382	0.24	−0.6	−0.2	−0.44	92	93	−0.47
(0, 1, 4)	99.6099	99.6096	0.32	−0.4	0.1	−0.50	97	98	−0.20
(1, 1, 4)	103.6283	103.6286	−0.24	0.4	0.4	0.01	105	103	0.66
(1, 3, 3)	107.7175	107.7176	−0.17	0.4	0.7	−0.29	108	109	−0.46
(0, 2, 4)	111.9024	111.9021	0.26	0.5	1.0	−0.50	117	116	0.48
(1, 2, 4)	116.2141	116.2143	−0.16	0.9	1.2	−0.27	123	124	−0.33
(2, 3, 3)	120.6926	120.6929	−0.34	1.5	1.6	−0.08	131	133	−0.76
(2, 2, 4)	130.3809	130.3808	0.12	2.2	2.6	−0.43	157	160	−0.70
(0, 0, 5)	135.7736	135.7739	−0.29	3.0	3.1	−0.18	177	179	−0.82
(1, 3, 4)	141.7506	141.7510	−0.34	3.7	3.9	−0.24	205	208	−0.78
(3, 3, 3)	148.6569	148.6572	−0.27	5.0	5.3	−0.29	255	254	0.20

## Footnotes

1 Certain commercial equipment, instruments, or materials are identified in this paper in order to specify the experimental procedure adequately. Such identification is not intended to imply recommendation or endorsement by the U.S. government, nor is it intended to imply that the materials or equipment identified are necessarily the best available for the purpose.

2 The Fundamental Parameters Approach Python Code (FPAPC) is provided by NIST as a public service. The FPAPC is provided for research purposes only and is not to be used for commercial purposes. Use of the FPAPC is subject to the terms and conditions which accompany the FPAPC itself.

## 5. Appendix A. Convolutions in Fourier Space

A computational technique, such as the FPA, which depends heavily on convolutions often can be represented very efficiently in Fourier space via the Convolution Theorem. That is, if a convolution is written as:

$$f\left(x\right)⊗g\left(x\right)\equiv \int_{-\infty }^{\infty }f\left(x-x^{\prime }\right)g\left(x^{\prime }\right)dx^{\prime }$$ (40)

and if we use $\tilde{f}$ to represent the Fourier transform of *f*, then

$$\tilde{f⊕g}=\tilde{f}\tilde{g}$$ (41)

which is to say that a convolution reduces to multiplication of Fourier transforms. If one is working with functions on a discrete grid, computing the convolution directly from 40 may be an efficient technique if the number of non-zero elements in *g* is much less than the number of elements of *f*. The time to carry out a convolution directly scales as *n_f_n_g_* where the *n* are the length of the sets. However, using FFT only costs a time proportional to *n* log *n*, so if log *n* is of the order of the length of *n_g_* or less, it may be advantageous to work in Fourier space. Since it is often the case that log *n* ≪ *n_g_*, and the penalty in the other case is fairly minimal, we choose to do all our convolution work in Fourier space. As such, we will derive analytic forms for the Fourier transform of most of the functions we need, and avoid transforming real-space functions into Fourier space to carry out the convolutions. This is not a major factor in the present paper. We compute the Axial Divergence function in real space, because it is much more easily represented there than in Fourier space, and then take the FFT of it to perform the convolution. All the other convolutions could be equivalently computed and carried out in real space.

Some important cases of analytic forms of transforms we use are presented in this section.

### 5.1 Top-Hat Function

If *f* (*x*) = 1/*w* for −*w*/2 < *x* < *w*/2 (a unit-area top hat), then

$$\tilde{f}\left(\omega \right)=\frac{\sin\left(w\omega /2\right)}{w\omega /2}\equiv \text{sinc}\left(\frac{w\omega }{2\pi }\right)$$ (42)

where the sinc function is defined as (in some implementations)

$$\text{sincx}=\frac{\sin\pi x}{\pi x}.$$

One should check if using sinc since another standard omits the *π*. It is useful to use instead of directly computing the ratio, since the ratio produces a 0/0 at the origin, and the sinc function correctly takes the limit.

### 5.2 Gaussian Function

If *f* (*x*) is the unit-area Gaussian as follows:

$$f\left(x\right)=\frac{1}{\sqrt{2\pi \sigma }}\exp\left(-\frac{x^{2}}{2\sigma^{2}}\right)$$ (43)

then its Fourier transform is:

$$\tilde{f}\left(\omega \right)=\exp\left(-\frac{x^{2}\sigma^{2}}{2}\right).$$ (44)

Also worth noting is that in X-ray diffraction, a Gaussian is often specified by its FWHM. *σ* is related to the FWHM *s* as

$$\sigma^{2}=\frac{s^{2}}{8\log2}.$$ (45)

### 5.3 Lorentzian Function

If *f* (*x*) is the unit-area Lorentzian with FWHM Γ, as follows:

$$f\left(x\right)=\left(\frac{\Gamma }{2\pi }\right)\frac{1}{x^{2}+{\left(\Gamma /2\right)}^{2}}$$ (46)

then its Fourier transform is:

$$\tilde{f}\left(\omega \right)=\exp\left(-\left|\Gamma \omega /2\right|\right).$$ (47)

### 5.4 One-Sided Exponential Tail

If *f* (*x*) =*δ* exp(*δx*) for *x*_0_ < *x* < 0 (with *x*_0_ < 0) and zero elsewhere, then the Fourier transform of the function is

$$\tilde{f}\left(\omega \right)=\frac{1}{\delta }\frac{1-\exp\left(x_{0}\left(i\omega +1/\delta \right)\right)}{i\omega +1/\delta }.$$ (48)

Note that we present this in not-quite-normalized form. If *x*_0_ → −∞, it has unit area. We will use this in the case of finite sample thickness, in which case it should not be normalized to unit area, but just as shown here.

### 5.5 Translation of a Function

The Fourier transform of *f* (*x* + *a*) for any *f* is:

$$\tilde{f\left(x+a\right)}=\tilde{f}\left(\omega \right)\exp\left(-i\omega a\right).$$ (49)

## 6. Appendix B. Mapping of Important Variables from Sample Python Code to Paper, and Other Code Notes

**Code available at http://dx.doi.org/10.6028/jres.120.014.c**

### 6.1 Names

**Python name** name in paper and explanation

**axial_helper** *F*_0_()

**_fxbuf** no name in paper, a scratch buffer saved for memory efficiency

**searchsorted()** a function which takes an ordered array and a list of values, and returns the bin index from the array for the number exactly matching each of the values, or returns the index of the bin to the right if the value falls between to bins.

### 6.2 Python Array Indexing

The most complex part of an efficient implementation of the FPA is the assignment of data to specific bins in a discretized representation of a computed diffraction profile. This is the reason for the complexity of *F*_0_. Because every computer language has its own conventions for how to index an array, and this is central to this work, we include here short notes about the Python language array indexing, to assist in translation to other languages. Thus, Python arrays:

- are indexed in a zero-based manner; x[0] is the first element of an array
- permit negative indexing to index from the last element; x[−1] is the last element of an array
- can have a sub-array taken from them by indexing x[1:5]; this means the four elements of the array x[1], x[2], x[3], and x[4]. The length of a slice is the difference between the ending and starting indices. This is a feature of python indexing which can cause confusion, but is quite handy. This rule also implies that x[1: −1] indexes everything except the first and last elements of the array.
- can have every second (e.g.) element, starting from 1 and ending before 9, indexed as x[1:9:2]; this extracts x[1], x[3], x[5], and x[7]. Note that x[9] is excluded since the upper end is never included. Thus, to negate all the odd-numbered elements of an array, x[1::2]*= −1. Note that empty index slots mean all possible, so this includes (potentially) the last element of the array. This is used extensively to re-phase Fourier transforms so that the zero position of the inverse transform is in the center of an array, rather than at the left edge, with negative positions wrapped to the right edge.

### 6.3 General Notes

The code, as provided in the supplementary material, is quite a bit more complex than a stripped-down reference implementation normally would be. This is due to the fact this version includes quite a bit of capability related to caching results which are likely to be re-used in the process of least-squares fitting of peaks. It is advised that the reader wishing to understand the underlying theory pay primary attention to the contents of the “conv_xxx” functions, which contain the machinery used to generate the convolutions, and to the various functions beginning with “axial_” which handle the real-space parts of the axial divergence integral.
